# Research Progress and Critical Challenges of Bioartificial Kidneys in Renal Replacement Therapy for End-Stage Renal Disease

**DOI:** 10.3390/biom16081161

**Published:** 2026-08-10

**Authors:** Luoyi Chen, Qiang Zhang, Yizhong Tu, Tong Chen, Yunliang Xie, Kaixin Lan, Wei Yan, Chunyuan Xue, Shuangjin Yu, Jiang Qiu

**Affiliations:** 1 Organ Transplantation Center, The First Affiliated Hospital, Sun Yat-sen University, Guangzhou 510080, China; 2 Guangdong Provincial Key Laboratory of Organ Donation and Transplant Immunology, The First Affiliated Hospital, Sun Yat-sen University, Guangzhou 510080, China; 3 NHC Key Laboratory of Clinical Nephrology, Guangdong Provincial Key Laboratory of Nephrology, The First Affiliated Hospital, Sun Yat-sen University, Guangzhou 510080, China; 4 ARM Kidney Regeneration & Rebuilding Joint Lab, Sun Yat-sen University, Guangzhou 510080, China

**Keywords:** chronic kidney disease, bioartificial kidney, engineered filtration membrane, bioreactor, renal cell therapy

## Abstract

Chronic kidney disease (CKD), one of the major global public health burdens, continues to exhibit a rising prevalence worldwide. The growing population of patients with end-stage renal disease (ESRD) has led to an increasing demand for renal replacement therapy (RRT). Although dialysis effectively prolongs survival, it fails to fully replicate kidney function. In addition, the persistent shortage of donor kidneys results in prolonged waiting periods for kidney transplantation. Emerging renal replacement strategies, such as kidney organoids, have demonstrated considerable potential. However, multiple technical limitations continue to hinder their near-term clinical translation. Bioartificial kidneys (BAKs), which integrate engineering and biological technologies, generally consist of artificial filtration membranes and living-cell bioreactors designed to mimic native kidney function. Advances in nanotechnology, biomaterials, and tissue engineering have accelerated the development of implantable bioartificial kidneys (iBAKs), making them an important research direction in renal replacement therapy. These innovations have improved membrane performance, biocompatibility, and cellular integration; however, substantial challenges remain regarding long-term stability, immune compatibility, and clinical validation before translation into human applications. Specifically, limited cell sources and uncertain long-term biocompatibility remain major barriers to iBAK development. In the future, biosensors and artificial intelligence (AI) technologies may be incorporated into bioartificial kidneys to enable personalized precision therapy. This review focuses on the developmental and major challenges of bioartificial kidneys, with detailed discussion of recent progress in implantable artificial kidneys.

## 1. Introduction

Chronic kidney disease (CKD) is defined as persistent kidney damage or a progressive decline in kidney function lasting for at least three months, regardless of the underlying cause. It is recognized as an independent risk factor for cardiovascular disease and cognitive dysfunction. Population aging and the rising incidence of metabolic disorders have made CKD a major global public health challenge [1]. Recent epidemiological estimates from 2024 to 2025 suggest that CKD affects over 850 million individuals [2] globally and contributes to approximately 1.5 million deaths each year [3]. Among Chinese adults, the prevalence of CKD is estimated to be 8.2–10.8% [4]. However, despite the substantial patient population, awareness of the disease remains remarkably low at only 10% [5]. Due to delayed diagnosis and missed opportunities for early intervention, many patients eventually progress irreversibly to end-stage renal disease (ESRD). Globally, end-stage renal disease (ESRD) is estimated to affect over 4.59 million individuals and imposes a substantial healthcare and socioeconomic burden, accounting for approximately 2–4% of national healthcare expenditures in some countries. ESRD also contributes significantly to the global burden of disability-adjusted life years (DALYs) [6,7]. As patients progress to irreversible loss of kidney function, long-term renal replacement therapy (RRT) becomes essential for survival and places considerable demands on healthcare systems.

### 1.1. Dialysis and Kidney Transplantation

Currently, the demand for renal replacement therapy is substantial worldwide. In 2017, the global ESRD population reached approximately 5.3 million, and dialysis and kidney transplantation remained the main therapeutic strategies. Despite advances in dialysis technology, long-term outcomes remain unsatisfactory, with reported 5-year survival rates of approximately 42% in the United States and 33% in China among dialysis patients [8]. Moreover, dialysis patients experience a markedly increased risk of cardiovascular complications, infection, malnutrition, anemia, and osteoporosis, which collectively contribute to substantial morbidity and mortality [9,10]. According to the United States Renal Data System (USRDS) 2023 Annual Data Report, cardiovascular events, including arrhythmia and cardiac arrest, accounted for 44.0% of known causes of death among hemodialysis patients [11]. Beyond clinical complications, dialysis imposes considerable socioeconomic pressure, with Medicare expenditures for ESRD patients reaching $55.3 billion in 2023 according to the USRDS 2025 Annual Data Report [12]. Furthermore, the repetitive and restrictive nature of dialysis negatively affects patients’ quality of life and psychological well-being, with depression reported in 10–45% of hemodialysis patients [13]. Compared with dialysis, allogeneic kidney transplantation provides superior long-term survival outcomes. However, the extreme shortage of donor kidneys severely limits its availability [14]. The kidney transplantation rate among dialysis patients was only approximately 4 transplants per 100 people in 2024 [12]. Furthermore, among patients who remained on the kidney transplant waiting list for 5 years, 18.5% died before receiving a transplant [15]. These limitations highlight the urgent need for alternative renal replacement strategies capable of providing more physiological, continuous, and sustainable kidney support.

### 1.2. Emerging Strategies for Renal Replacement Therapy

Rapid advances in regenerative medicine, bioengineering, and genome editing have driven the emergence of novel renal replacement strategies, including decellularized kidney scaffolds, human pluripotent stem cell-derived kidney organoids, interspecies chimeras, and xenotransplantation [16,17]. Although early studies have demonstrated the regenerative potential of kidney organoids and interspecies chimeras [18,19,20,21,22], inadequate cellular maturation, limited microvascularization, and low in vivo chimeric efficiency continue to impede clinical translation [23,24,25]. In contrast, xenotransplantation has advanced more rapidly toward clinical application, with multigene-edited and humanized donor pigs enabling its entry into clinical trials [26]. Meanwhile, exploratory xenotransplantation studies in China have further demonstrated the preliminary feasibility of multigene-edited donor organs and novel immunosuppressive strategies in the human microenvironment [27,28]. Incomplete physiological compatibility, particularly in coagulation pathways, together with the potential cross-species transmission of porcine endogenous retroviruses (PERVs), continues to raise safety concerns [29,30]. Ethical concerns regarding gene editing and interspecies transplantation further complicate the widespread clinical application of xenotransplantation [31].

**Figure 1:**
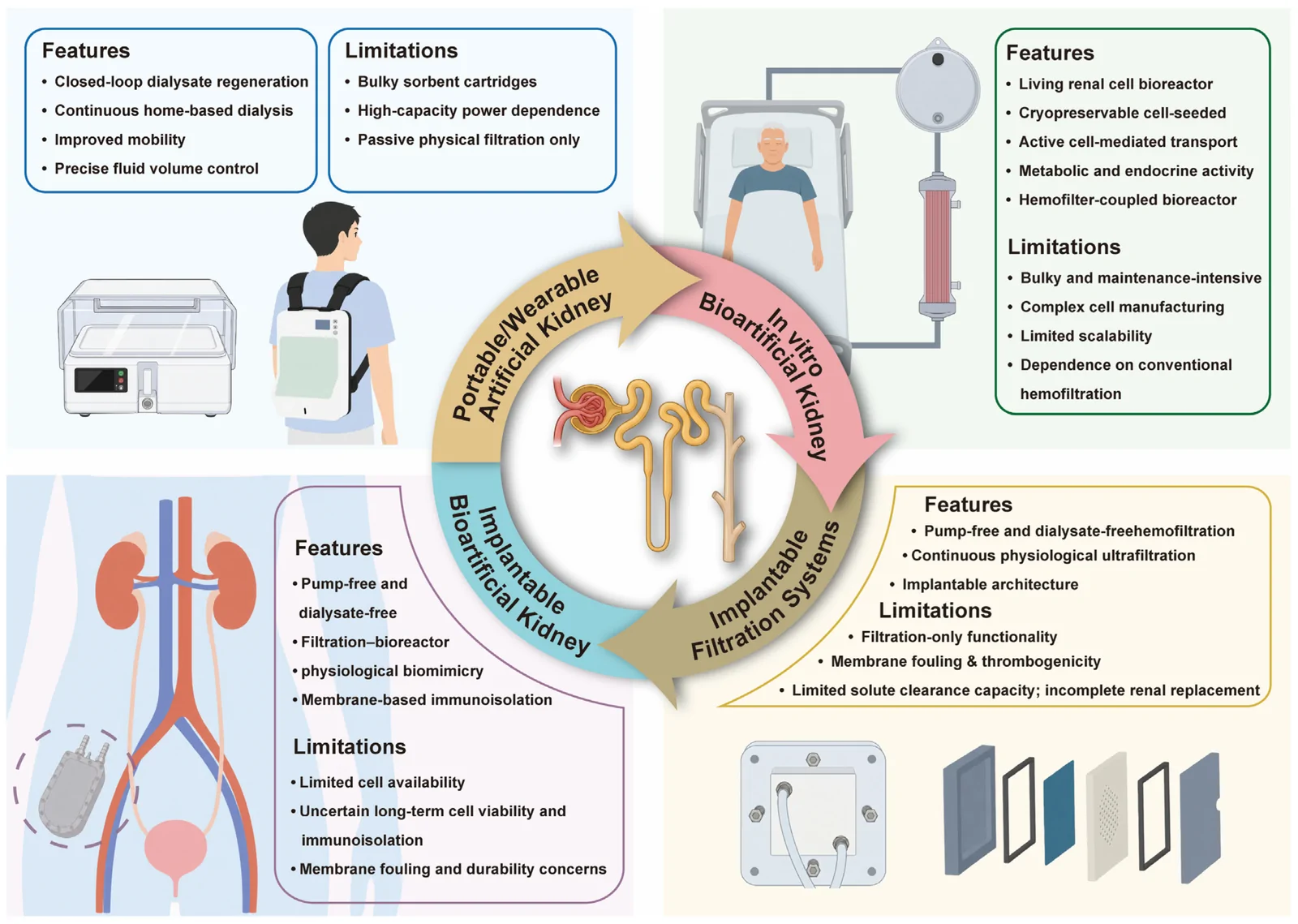
Development, classification, and key characteristics of artificial kidney technologies. Artificial kidneys have evolved from portable dialysis devices toward implantable and biohybrid systems that increasingly mimic native renal function. Four major categories are highlighted. Portable/wearable artificial kidneys improve mobility through device miniaturization but remain constrained by dependence on dialysate regeneration. In vitro bioartificial kidneys combine hemofilters with living renal cell bioreactors to restore tubular, metabolic, and endocrine functions, although complex cell manufacturing and scalability remain challenges. Implantable filtration systems provide pump-free, dialysate-free hemofiltration driven by physiological blood pressure, yet are limited to incomplete renal replacement. Implantable bioartificial kidneys represent the most advanced biohybrid concept but continue to face obstacles related to cell sourcing, immunoisolation, and long-term durability.

Given the persistent shortage of donor kidneys and the limitations of xenotransplantation, bioartificial kidneys (BAKs) have emerged as a promising alternative with the potential to provide physiological replacement while reducing immune and infection-related risks. Modern artificial kidney research is evolving toward device miniaturization and physiological biomimicry to overcome the limitations of conventional dialysis. Clinically, artificial kidney technologies are currently being developed toward two major therapeutic applications: temporary renal support for acute conditions and long-term renal replacement for chronic kidney failure. Acute support strategies primarily rely on extracorporeal systems integrated with hemofiltration or continuous renal replacement therapy (CRRT) to provide short-term metabolic regulation and immunomodulatory effects in critical conditions with acute kidney injury (AKI), sepsis, or multiorgan failure. Conversely, chronic replacement strategies aim to achieve sustained restoration of renal function, with the potential to complement or ultimately replace conventional maintenance dialysis and kidney transplantation. Within this long-term chronic landscape, current artificial kidney engineering is primarily advancing along three major directions. Portable or wearable artificial kidneys incorporate closed-loop dialysate regeneration technologies to reduce reliance on external water supplies and facilitate continuous home-based dialysis. The second is fully implantable mechanical filtration systems. However, both extracorporeal wearable devices and purely mechanical implantable systems rely on passive physical filtration, limited by their inability to replicate the metabolic and endocrine functions of native kidneys. These limitations of purely mechanical systems have accelerated the development of BAKs, integrating advanced filtration membranes with living-cell bioreactors. Although substantial progress has been achieved in artificial kidney engineering, current studies remain distributed across diverse technological platforms, including extracorporeal bioartificial systems, wearable and portable artificial kidneys, implantable mechanical devices, and implantable bioartificial kidneys (iBAKs). A comprehensive overview integrating recent advances, technical challenges, and translational barriers is therefore needed to better understand the developmental trajectory of artificial kidney technologies and evaluate their potential role in next-generation renal replacement therapy. In this review, we summarize recent progress in various artificial kidney systems, with particular emphasis on their design principles, functional characteristics, limitations, and future perspectives toward clinical translation (Table 1 and Figure 1).

## 2. Methods

This narrative review was based on a comprehensive search of the published literature and publicly available technical information. Relevant studies were identified from PubMed, Web of Science, and Scopus databases from inception to April 2026 using combinations of keywords including “artificial kidney”, “bioartificial kidney”, “wearable artificial kidney”, “portable artificial kidney”, “implantable bioartificial kidney”, “renal replacement therapy”, “hemofiltration”, “renal assist device”, “bioartificial renal epithelial cell system”, “kidney tissue engineering”, and “kidney organoid”. Clinical trial information was additionally retrieved from ClinicalTrials.gov, while patent databases, regulatory documents, and official technical reports were consulted to identify emerging device designs and technological developments. Articles and information sources were selected based on their relevance to artificial kidney development, including engineering principles, biological components, preclinical and clinical evaluations, and translational challenges. Non-English publications, unrelated studies, and sources lacking sufficient scientific or technical relevance were excluded.

## 3. Current Status of Bioartificial Kidneys and Advanced Systems

BAKs represent an emerging renal replacement strategy intended to supplement or potentially replace conventional dialysis and kidney transplantation [81,82]. By integrating engineering and biotechnology, BAKs are designed to mimic native kidney function while improving portability or implantability, thereby enhancing both therapeutic efficacy and quality of life in patients with ESKD. BAKs generally comprise an engineered filtration membrane with a cell-containing bioreactor to integrate mechanical filtration and biological activity [83], which could provide broader renal functions including reabsorption, metabolic regulation, and endocrine activity, potentially improving patient quality outcomes. According to their intended clinical applications, current BAK platforms can be broadly categorized into systems designed for acute kidney injury (AKI) or temporary extracorporeal renal support, and systems aiming for continuous renal replacement in chronic kidney failure. Early BAK development primarily relied on proximal tubular cells cultured on artificial membranes to reproduce renal tubular functions in vitro, exemplified by the renal assist device (RAD) and bioartificial renal epithelial cell system (BRECS). These systems utilize extracorporeal biohybrid platforms to provide short-term metabolic regulation and immunomodulatory effects in critically ill patients. With advances in materials science, nanotechnology, and bioengineering, BAK development is currently shifting toward more multidisciplinary, miniaturized, and implantable systems which focus on achieving sustained renal replacement [84,85]. Accordingly, implantable bioartificial kidneys (iBAKs) have become a major research focus, with several systems already entering preclinical studies.

### 3.1. Systems for Acute Kidney Injury (AKI) or Temporary Extracorporeal Support

In the intensive care unit, severe acute kidney injury (AKI) may progress to systemic inflammatory response syndrome (SIRS), sepsis, and multiple organ dysfunction syndrome (MODS). Although continuous renal replacement therapy (CRRT) provides fluid and small-solute clearance, it remains a passive filtration modality lacking renal parenchymal cell-mediated functions and immunomodulatory capacity. To overcome these limitations, early BAK systems incorporated living renal cells into extracorporeal platforms to provide temporary metabolic, transport, and immunomodulatory support until recovery of native kidney function. These pioneering efforts established the technological and biological foundation for the subsequent development of implantable bioartificial kidney (iBAK) systems.

#### 3.1.1. Renal Assist Device

The concept of integrating hemofiltration devices with renal tubular cells in BAKs was first proposed in 1987 by Aebischer et al. [86]. In 1989, renal epithelial cell monolayers were successfully cultured on permeable substrates with different physicochemical properties, providing the foundation for subsequent cell-based renal replacement strategies [86]. Based on this concept, H. David Humes et al. developed the renal assist device (RAD) in 1998 by seeding human proximal tubular cells onto the inner surface of polysulfone hollow-fiber hemofiltration cartridges, generating a confluent epithelial monolayer containing up to 10^9^ cells [38]. In vitro studies demonstrated that RAD enabled vectorial transport across renal epithelial cells and preserved key tubular metabolic and endocrine functions, including bicarbonate and glucose transport, glutathione metabolism, and vitamin D activation [33,38]. Preclinical large-animal studies further showed that RAD combined with extracorporeal hemofiltration could partially restore renal filtration, transport, metabolic, and endocrine functions in uremic canine models and attenuate endotoxin-induced shock [36,37].

RAD subsequently became the first cell-based BAK platform to enter clinical evaluation. In Phase I/II clinical studies, RAD was safely applied for 24 h in 10 patients with AKI undergoing continuous veno-venous hemofiltration (CVVH), with a reported reduction in hospital mortality of 40–55% [87]. Furthermore, a multicenter randomized phase II trial demonstrated a reduction in 28-day mortality from 61% in patients receiving conventional CRRT to 33% in those treated with RAD [34]. However, the subsequent phase IIb clinical trial evaluating commercial-scale development of RAD was discontinued after interim analysis because the study was unlikely to demonstrate the predefined primary efficacy endpoint. Subsequent analyses suggested that the unexpected improvement in the sham device group substantially reduced the apparent incremental benefit attributable to the renal tubular cell component. Importantly, the sham device should not be considered a completely inert control, as the hollow-fiber membrane system combined with citrate anticoagulation was later found to exert immunomodulatory effects, including modulation of activated inflammatory cells, which may have contributed to improved outcomes in the control group. This experience highlighted an important consideration for clinical trial design of combination bioengineered products: the biological effects of the device platform itself must be carefully evaluated when assessing the additional therapeutic contribution of cellular components [88].

Beyond clinical trial design, the RAD program also revealed major translational barriers associated with the development of cell-based renal replacement therapies. The reliance on primary human renal proximal tubular cells (HPTCs) posed significant challenges in cell sourcing, large-scale manufacturing, and maintenance of stable cellular phenotypes. In addition, preserving cell viability and functional polarization within a complex extracorporeal device, together with requirements for cell expansion, storage, transportation, and standardized clinical operation, created substantial logistical and economic obstacles that limited scalability and commercialization. These lessons have subsequently guided the development of next-generation BAK platforms, including alternative cell sources, scalable cell-delivery systems such as BRECS [42], and implantable bioartificial kidneys with improved manufacturing feasibility and long-term clinical applicability.

#### 3.1.2. Bioartificial Renal Epithelial Cell System

To overcome cell sourcing limitations, bioartificial renal epithelial cell system (BRECS) researchers isolated human renal epithelial cells (HRECs) from donor kidneys unsuitable for transplantation. They expanded renal progenitor cells using the enhanced propagation (EP) technique which increased cell yield by 108-fold while outperforming cells used in RAD trials in terms of metabolic activity and cell phenotype. The expanded cells were seeded onto niobium-coated carbon disks with favorable thermo-mechanical properties and maintained viability under perfusion culture. All BRECS components are compatible with cryopreservation, enabling long-term storage at −140 °C and short-term storage at −80 °C. After thawing, BRECS cells were shown to retain metabolic and phenotypic stability, with viability loss remaining below 10% [42], and maintained viability in both the septic shock (SS) pig model and the chronic uremic sheep model. Additionally, in SS pig models, BRECS integrated with CVVH significantly improved cardiovascular performance, reduced inflammatory cytokine levels, and prolonged survival [41].

BRECS was the first platform to combine cell expansion, cryopreservation, and therapeutic delivery into a single compact system. This design supports scalable manufacturing and clinical distribution and may facilitate combination with wearable dialysis systems for in vitro kidney replacement therapy [40]. However, in vitro BAK systems still depend on external dialysate and power supplies and remain bulky and maintenance-intensive. In response to these limitations, iBAKs have been conceptualized as an advanced alternative intended to eliminate reliance on external dialysate and power systems, thereby potentially enhancing future clinical applicability. The explorations of RAD and BRECS have provided preliminary insights into the design of iBAKs.

### 3.2. Systems for Continuous Replacement in Chronic Kidney Failure

For long-term management of chronic kidney failure, ideal renal replacement systems should provide continuous restoration of kidney function while maintaining fluid and electrolyte homeostasis with minimal impact on patients’ quality of life. Early engineering efforts have focused on WAKs and AWAKs, which employ closed-loop dialysate regeneration technologies to improve mobility and enable more flexible dialysis schedules. However, these systems remain limited by device complexity, size constraints, and their inability to restore the metabolic and endocrine functions of native renal tissue. In 2013, Shuvo Roy from the University of California, San Francisco proposed the concept of iBAK. The system integrates a hemofilter and bioreactor in series and connects directly to the iliac vasculature and bladder. Ultrafiltration is driven by the patient’s own blood pressure, eliminating the need for external pumps and dialysate. Concentrated waste is discharged into the bladder. Since the introduction of iBAK, miniaturized hemofilters and bioreactors have become major research focuses.

#### 3.2.1. Advanced Wearable and Portable Artificial Kidneys

To overcome the limitations of intermittent center-based hemodialysis in patients with chronic kidney failure, continuous and portable renal replacement strategies have been developed to improve treatment flexibility and patient autonomy. Wearable artificial kidneys (WAKs), including the automated wearable artificial kidney (AWAK) and other sorbent-based dialysis systems, represent early efforts toward miniaturizing conventional dialysis and enabling home-based continuous therapy. These platforms have provided important engineering foundations for subsequent implantable systems by advancing technologies related to dialysate regeneration, device miniaturization, fluid management, and long-term extracorporeal operation. However, challenges in achieving sufficient clearance capacity, maintaining device durability, and ensuring long-term biocompatibility have limited their clinical translation.

Building upon these advances, implantable continuous mechanical filtration systems have emerged as a further step toward autonomous renal replacement. The Holly system, developed by Nephrodite, integrates an implantable continuous hemofiltration unit, intelligent sensors, machine-learning-based monitoring, and an external component for supplemental dialysis support. In a 72 h sheep study, the system achieved stable clearance of urea, creatinine, and potassium while maintaining fluid balance without thrombosis or hemolysis, and subsequently received FDA Breakthrough Device Designation [63,64]. Nevertheless, similar to WAKs, these mechanical systems primarily replace glomerular filtration and solute removal but cannot reproduce renal tubular metabolic and reabsorptive functions or endocrine activities [89,90]. These limitations have driven the transition from purely mechanical replacement toward implantable bioartificial kidneys (iBAKs), which aim to integrate advanced filtration technologies with living-cell-based renal functions.

#### 3.2.2. Pump-Free Implantable Hemofiltration Devices

Even though the ultimate goal of iBAKs is cellular-level functional replacement, a major engineering challenge for iBAKs is maintaining stable pump-free hemofiltration only through arteriovenous pressure differences without inducing backfiltration driven by oncotic pressure. Recent studies have developed pump-free microfluidic hemofiltration systems driven entirely by pressure difference between the artery and the vein. To prevent backfiltration driven by transmembrane pressure (TMP), the system was divided into a hemofiltration module composed of polyethersulfone nanoporous membranes and a serial fluid-resistive module which could maintain positive TMP by generating a controlled pressure drop, thereby preventing backfiltration. In vitro and in vivo studies demonstrated that this microfluidic device could sustain stable ultrafiltration and toxin clearance for up to 90 min without external power [58]. More recently, a hybrid renal replacement strategy incorporating an implantable hemofiltration device was further validated in a 10-day goat experiment, in which continuous filtrate production was successfully maintained under natural blood pressure without an external pump, supporting the feasibility of prolonged in vivo operation [61]. This pump-free miniaturization strategy, particularly its fluid dynamic regulation of transmembrane pressure to prevent backfiltration, provides valuable engineering insights for the future design of iBAKs.

#### 3.2.3. Silicon Nanopore Membranes-Based iBAK Bioreactor

Nanotechnology, widely applied in miniaturized implantable medical devices, primarily involves the design and manipulation of materials and devices at the 1–100 nm scale through precise control of atomic and molecular structures. Microelectromechanical systems (MEMSs) are miniaturized platforms integrating mechanical components, electronic elements, and sensors on a single chip, typically at the millimeter or micrometer scale. Owing to their compact and integrated design, MEMS devices are widely used in wearable and self-powered medical systems, with silicon serving as the primary fabrication material [91]. Shuvo Roy et al. first investigated silicon nanopore membranes (SNMs) for blood filtration in 2009. SNMs exhibit favorable biocompatibility and mechanical durability. Compared with conventional dialysis membranes, MEMS-fabricated SNMs have slit-shaped pore architectures that resemble the native glomerular filtration barrier, which allows for selective toxin removal with lower flow resistance, less protein loss, and better bioreactor protection from immune cell infiltration [66]. Additionally, surface modification of SNMs with polymers, such as polyethylene glycol and polyvinylamine, could inhibit platelet adhesion and protein adsorption, thereby enhancing hemocompatibility [65,67,71]. Studies demonstrated that SNMs coated with ultrathin (<5 nm) polysulfobetaine methacrylate (pSBMA) exhibited enhanced resistance to shear stress and minimal platelet adhesion and activation in both 7-day in vitro blood circulation experiments and 26-day porcine implantation models. Preclinical studies showed that implantable SNM filters achieved effective solute clearance (e.g., potassium, creatinine, and blood urea nitrogen) and albumin preservation without immunosuppression or major adverse events in canine and porcine models [70,92].

Despite these advances, implantable SNM filters provide only hemofiltration and must be combined with renal cellular bioreactors to achieve full kidney replacement function. In 2023, Shuvo Roy and William H. Fissell launched a nationwide collaborative kidney project, aiming to integrate SNMs with HREC bioreactors to develop self-regulating implantable iBAKs for continuous renal replacement therapy. In a feasibility study, an iBAK prototype was constructed and implanted, comprising vascular flow pathways and an SNM-integrated bioreactor. The bioreactor was assembled by seeding HRECs onto the cell inserts stacked with SNMs, and optimized blood flow path by computational fluid dynamics. Seven-day porcine implantation studies showed that SNMs could protect HRECs from inflammatory injury without systemic anticoagulation or immunosuppression, preserving approximately 90% viability, transporter gene expression and vitamin D activation. In addition, an optimized blood flow path could minimize thrombosis and protein fouling, maintaining device patency and function after implantation [74]. These findings preliminarily support the feasibility of iBAKs without systemic immunosuppression, although long-term durability and functional performance remain to be established. Future studies will focus on increasing cellular capacity and implantation duration to further evaluate the potential for clinical application.

#### 3.2.4. Ceramic Membrane-Based iBAK Bioreactor

Ceramic membranes are inorganic porous membranes commonly composed of Al_2_O_3_, ZrO_2_, or TiO_2_, and are widely used in biomedical and industrial filtration applications. Bioceramics are widely used in dental and orthopedic implants owing to their favorable mechanical and biological properties [93,94,95]. These membranes exhibit highly uniform pore distributions and precise nanoscale filtration capability, allowing efficient clearance of toxins and metabolic waste from blood. In addition, their high mechanical strength and chemical stability enable them to support durable filtration performance under high transmembrane pressure and complex operating conditions, and their antifouling properties can reduce protein adsorption and membrane fouling. Compared with silicon-based nanoporous membranes, which often require additional surface modification strategies, such as protein coatings, to improve cell adhesion and preserve tubular epithelial phenotype under shear stress, bioceramic membranes provide a mechanically robust and chemically stable scaffold with inherent biocompatibility [96]. These characteristics may endow bioceramic membranes with a long service life and low maintenance frequency and reduce the dependence on complex surface modification while supporting long-term integration of renal cells, offering significant cost-effectiveness advantages. Nevertheless, despite these advantages, material brittleness and manufacturing complexity remain important limitations that require further optimization during the development of bioceramic membrane-based iBAKs.

In a novel iBAK project, bioceramic membranes and renal cell perfusion systems were integrated to establish selective filtration and reabsorption modules in a novel iBAK platform. This bioceramic membrane features adjustable pore sizes with a uniform size distribution, controllable thickness, and low protein adsorption. In preliminary experiments, the membrane demonstrates favorable biocompatibility, high toxin clearance, and low thrombogenicity. Furthermore, perfused renal cells formed highly confluent monolayers on the membrane surface and demonstrated selective reabsorptive capability. This iBAK device employed flexible housing integrating interstitial buffering, high-efficiency filtration, and biological modules, with artificial vascular and ureteral conduits connecting the device to the iliac vessels and bladder. In AKI animal models, this iBAK device significantly reduced inflammatory cytokines and exhibited improved toxin clearance and partial selective reabsorptive function [80,97].

#### 3.2.5. Hydrogel-Based iBAKs

Beyond nanotechnology and MEMSs, 3D printing has emerged as an important tool in biomedical engineering. 3D printing is an additive manufacturing approach that converts digital three-dimensional models into highly precise and customizable physical structures [98]. In tissue engineering and regenerative medicine, 3D bioprinting is considered a promising approach to address donor organ shortages. Living cells are employed by bioprinting to construct functional tissues and organ-like structures [99,100]. In this process, biocompatible scaffold design is essential for creating a favorable microenvironment that promotes renal cell growth, differentiation, and functional maturation, thereby facilitating renal tissue regeneration [101]. Hydrogels are widely used scaffold materials that support cellular attachment and differentiation owing to their favorable biocompatibility, bioactivity, high water content and hydrophilic properties. In addition, their physicochemical and mechanical properties further enable the fabrication of complex three-dimensional architectures for biomedical applications [102].

IVIVA Medical focuses on renal engineering technologies, including bioreactors, organ culture platforms, and scaffold fabrication strategies. They developed a novel scaffold fabrication strategy, thin film interposition (TFI). TFI bypasses the resolution constraints of conventional 3D printing by generating hydrogel-based stacked hollow vascular networks, with both microscale and macroscale features. Primary cells isolated from discarded donor kidneys were seeded into TFI scaffolds together with other human primary cells to generate functional tissues with the potential for renal replacement. Large-animal studies demonstrated both perfusability and the preliminary safety of the device [75,76,77,78]. Nevertheless, detailed experimental data remain unavailable, and current evidence remains limited to preliminary large-animal safety studies. Further validation of renal functional performance is still required.

Collectively, current studies suggest that BAKs have demonstrated preliminary safety and feasibility in preclinical investigations, highlighting their potential as a next-generation approach for renal replacement therapy. However, most existing studies remain at the preclinical stage and primarily focus on proof-of-concept validation, device safety, and short-term functional assessment. Further investigations addressing long-term biocompatibility, sustained efficacy, functional durability, and clinical safety are required to facilitate the translation of BAK technologies into routine clinical practice. In addition, direct comparison among different BAK platforms remains difficult because functional evaluations have not been standardized and are often tailored to specific developmental objectives. Establishing unified assessment criteria encompassing filtration, transport functions, endocrine activity, immunomodulatory effects, and long-term stability will be essential for future translational studies.

## 4. Difficulties and Challenges in the Development of Bioartificial Kidneys

### 4.1. Cell Sourcing and Phenotypic Stability

Although bioartificial kidneys have advanced rapidly as a novel renal replacement strategy, substantial challenges remain. Limited cell sources and difficulties in large-scale pathogen-free cell culture remain major barriers to the commercialization and routine use of BAKs [88]. To date, human primary proximal tubular cells (HPTCs) remain the predominant cellular source for BAK bioreactors. Derived from diseased or otherwise non-transplantable kidneys, the biological function of HPTCs may be compromised by underlying pathology. Repeated passaging would also reduce epithelial stability and limit the long-term application of primary cells. To overcome these challenges, researchers utilized retroviral vectors to express the human telomerase reverse transcriptase (hTERT) transcription factor, thereby achieving the immortalization of HPTCs. While immortalization appears to preserve cellular differentiation and function, its long-term safety still requires further evaluation [103]. Induced pluripotent stem cells (iPSCs) also provide an alternative cell source with unlimited expansion and multilineage differentiation potential. In a recently developed differentiation protocol, iPSCs could differentiate into proximal tubular-like (PTL) cells within 14 days. These PTL cells could not only express proximal tubular-specific markers but also maintain stable polarized phenotypes after serial passaging. These findings suggest that iPSC-derived cells may serve as promising alternative cell sources for BAKs [104,105]. In addition to stable cellular sources, scalable low-cost manufacturing and rigorous quality control are also critical for clinical application and safety of BAKs.

### 4.2. Immunoisolation and Immune Compatibility

Another important concern for iBAKs is the long-term effectiveness of immunoisolation. Current iBAKs generally rely on allogeneic renal epithelial cells. Therefore, effective protection from host immune surveillance is essential. Similar challenges have also been recognized in earlier cell-based bioartificial kidney platforms, including the RAD and BRECS. These systems employed semipermeable membrane barriers to separate therapeutic renal cells from circulating blood while preserving cellular metabolic and endocrine functions. SNMs have shown promising immunoprotective potential. In vitro studies demonstrated that SNMs could prevent the diffusion of TNF-α. In vivo studies further showed that xenogeneic HRECs remained viable and functional after short-term implantation in immunocompetent pigs without systemic immunosuppression. However, these findings were obtained primarily from short-duration studies focused on hyperacute immune responses. The ability of SNMs to prevent chronic exposure to diverse immune mediators, including cytokines, antibodies, complement components, and cellular immune responses, remains insufficiently characterized. Therefore, long-term in vivo studies are still required to determine whether immunoisolation can be maintained over clinically relevant implantation periods while preserving stable cellular function.

### 4.3. Biocompatibility and Functional Durability

On the other hand, biocompatibility and long-term functional durability are fundamental requirements for all implantable medical devices, which are essential for minimizing repeated surgery and device-related complications [106]. Although current animal studies suggest that iBAKs are feasible, their long-term functionality and durability after implantation remain unclear. In addition, iBAKs demonstrated favorable biocompatibility and safety, particularly SNM-based iBAK bioreactors that provide short-term immunoprotection without thrombosis and protein fouling. However, progressive nanopore obstruction may compromise the long-term filtration of iBAKs. Most studies of iBAKs are limited by short experiment durations and small sample sizes. Therefore, the long-term performance of the device and the persistence of cellular function should be further evaluated through additional investigations.

### 4.4. Regulatory and Translational Challenges

Beyond the aforementioned biological and engineering challenges, regulatory approval represents an additional critical barrier limiting the clinical translation of BAKs. Unlike conventional dialysis devices, BAKs are complex biohybrid systems integrating filtration components, biomaterials, and living cellular elements, which introduces challenges in product classification, safety evaluation, and quality control [107]. Regulatory assessment requires robust strategies to ensure consistent cell sourcing, manufacturing standardization, long-term cellular viability, and functional stability. Moreover, immune compatibility remains a major concern for both allogeneic and xenogeneic cell-based approaches, including potential cross-species pathogen transmission. The lack of fully predictive large-animal models and standardized clinical endpoints further complicates the evaluation of long-term safety and efficacy before clinical trials [108]. Addressing these regulatory and translational challenges will be critical for establishing reliable evaluation frameworks and accelerating regulatory approval of BAK platforms, particularly iBAKs.

Collectively, the challenges associated with BAK development span multiple biological, engineering, and translational domains (Table 2). Among these, reliable cell sources, stable cellular phenotypes, effective immunoisolation, and long-term device safety represent critical challenges that directly influence the feasibility of first-in-human studies. Engineering optimization, including membrane fouling control, device durability, and real-time monitoring, remains essential for maintaining long-term functionality. In addition, regulatory evaluation, manufacturing scalability, and cost considerations will ultimately determine the successful clinical implementation of BAK platforms. Although several BAK-related technologies have demonstrated encouraging preclinical feasibility, only a limited number of BAK platforms have progressed toward clinical evaluation, highlighting the need for multidisciplinary strategies to overcome current translational barriers.

## 5. Perspectives

Designed as long-term renal replacement platforms, BAKs aim to recapitulate key physiological functions of the native kidney while reducing the limitations associated with conventional dialysis and organ transplantation. By minimizing dependence on external dialysate and mechanical components, implantable bioartificial kidneys (iBAKs) may offer improved mobility and treatment flexibility. However, further investigations are required to validate their long-term safety, functional durability, and clinical applicability before widespread translation.

To address the biological bottlenecks of cell sourcing and phenotypic variability detailed previously, future perspectives should prioritize the development of infinitely expandable, standardized cellular sources and culture protocols that ensure consistent cell production, stable adhesion, and sufficient nutritional support, thereby improving device reproducibility, reliability, and clinical applicability. Concurrently, resolving the critical challenges of immunoisolation and membrane fouling requires the continuous advancement of advanced nanomaterials that not only reduce protein fouling but also improve long-term immuno-protection and selective filtration without necessitating systemic immunosuppression.

From an engineering perspective, overcoming the limitations of long-term functional durability, shear stress, and safety relies on intelligent device integration. The incorporation of miniaturized biosensors into iBAKs will transcend current monitoring barriers by enabling real-time control of toxin clearance and ultrafiltration (UF) rates [106]. Furthermore, future interdisciplinary integration of microelectronics, communication technologies, and artificial intelligence (AI) will be essential. By continuously analyzing patients’ physiological indicators and device operating status in real-time, AI systems could dynamically adjust iBAK parameters to mitigate risks like transmembrane pressure (TMP) fluctuations and nanopore obstruction. Such intelligent management systems will optimize device performance, extend operational longevity, and ultimately facilitate personalized precision therapy and patient self-management [109,110,111,112].

During future clinical translation of BAK technologies, future clinical studies should evaluate not only technical feasibility and biological safety but also their comparative effectiveness against established renal replacement therapies, including dialysis and kidney transplantation. Such evaluations should incorporate clinically relevant endpoints, including patient survival, dialysis independence, hospitalization burden, treatment-related complications, and quality of life. Moreover, patient-reported outcomes and psychosocial assessments will be essential for determining the patient-centered benefits of BAKs beyond conventional efficacy measures. Prior to widespread clinical adoption, comprehensive health economic evaluations, including cost-effectiveness analyses, manufacturing scalability assessments, reimbursement strategies, and healthcare system implementation considerations, will be necessary to facilitate the sustainable integration of BAKs into future renal replacement care.

## 6. Conclusions

In conclusion, the escalating global prevalence of end-stage renal disease and the inherent limitations of conventional dialysis and organ transplantation underscore the urgent need for innovative renal replacement therapies. This review has systematically outlined the evolutionary trajectory of bioartificial kidneys, from early extracorporeal renal assist devices to implantable platforms (iBAKs). While iBAKs demonstrate immense potential in replicating the physiological, metabolic, and endocrine functions of the native kidney, their clinical translation remains constrained by critical biological, engineering, and regulatory challenges. Addressing the bottlenecks associated with scalable cell sourcing, long-term immunoisolation, functional durability, and standardized evaluation will require concerted, multidisciplinary efforts. Moving forward, the integration of advanced biomaterials, infinitely expandable cellular technologies, and AI-driven biosensors holds the promise of overcoming these translational barriers, ultimately paving the way for personalized, self-regulated, and sustainable renal replacement therapy.

## Figures and Tables

**Table 1:** Current progress of artificial kidney systems.

Intended Clinical Application	Technological Category	Device	Developers	Device Composition/Technical Features	Target Functions	Research Stage	Ref.
Acute Renal Support	In vitro Bioartificial	RAD	H. David Humes, David A. Buffington, William H. Fissell, et al.	Polysulfone hollow-fiber cartridge; confluent monolayers of human renal proximal tubule cells.	Vectorial fluid and solute transport; glutathione metabolism; calcitriol activation; systemic immunomodulation.	Clinical Phase II; (NCT00280072)	[32,33,34,35,36,37,38]
		BRECS	H. David Humes, Shuvo Roy, William H. Fissell et al.	Niobium-coated carbon disk; perfusion bioreactor; human renal progenitor cells; cryopreservable integrated system.	Supplemental metabolic, endocrine, and immunomodulatory renal replacement therapy.	In vivo preclinical (Sheep/Pigs)	[39,40,41,42]
Chronic Renal Replacement	Portable/ wearable Artificial Kidneys	WAK	Victor Gura et al.	Battery-powered pulsatile pump; high-flux polyarylethersulfone dialyzer; sorbent-based dialysate regenerator.	Continuous uremic toxin clearance; precise fluid volume control; electrolyte homeostasis.	Clinical (NCT02280005)	[43,44,45,46,47,48]
		Dialysate-free WAK	Xu Deng, Dehui Wang, Changsheng Zhao, et al.	Gas-permeable blood-repellent membrane; vapor-gradient phase-transition system; integrated adsorption module.	Dialysate-free water ultrafiltration; creatinine clearance; middle-molecule removal.	In vivo preclinical (Rabbits)	[49]
		AWAK	Marjorie W. Y. Foo, Sheena K. Gow, Htay, et al.	Sorbent-based tidal peritoneal dialysis system; single-lumen catheter; closed-loop multi-adsorber cartridge regeneration.	Continuous bloodless peritoneal dialysate regeneration; uremic solute clearance; fluid ultrafiltration.	Clinical (NCT03128073, NCT05827588)	[50]
		NeoKidney	NextKidney research team et al.	Sodium-neutral sorbent cartridge; carry-on-suitcase-sized closed-loop system; low-volume dialysate circuit.	Semi-continuous daily hemodialysis; isonatremic dialysate regeneration; high-efficiency uremic toxin clearance.	Clinical (NCT06024135)	[51,52,53]
		Wearable Filtrating Device	Chinese PLA General Hospital research team, et al.	Miniaturized hemofiltration circuit; lightweight battery-powered ultrafiltration pump; integrated safety alarm system.	Rapid relief of acute fluid overload; precise ultrafiltration; pulmonary edema prevention during emergency rescue.	Clinical (NCT05906355)	[54]
	Implantable Filtration Systems	IHFD	Yukihiko Kanno, Nobuhiko Miki, Takuya Ito et al.	Multilayered microfluidic system; flat-sheet polyethersulfone membrane; fluid-resistive channels preventing backfiltration.	Continuous fluid removal; pump-free hemofiltration; backfiltration prevention.	In vivo preclinical (Goat)	[55,56,57,58,59,60,61,62]
		Holly	Nephrodite research team et al.	Pelvic hemofilter; built-in monitoring sensors; portable tableside fluid-removal component.	Continuous daytime toxin clearance; nightly fluid removal; remote monitoring.	In vivo preclinical (Sheep)	[63,64]
	Implantable Bioartificial Kidney	SNMs-based iBAK Bioreactor	Shuvo Roy, William H. Fissell, H. David Humes et al.	Silicon nanopore membrane; PEG surface coating; encapsulated human renal epithelial cells.	Immunoprotection from host cytokines, selective solute reabsorption, metabolic endocrine homeostasis.	In vivo preclinical (Pigs)	[65,66,67,68,69,70,71,72,73,74]
		TFI-based iBAK	IVIVA Medical research team et al.	Thin Film Interposition hydrogel scaffold; vascular and epithelial micro-networks; patient-derived induced pluripotent stem cells.	Continuous biological blood purification, selective solute-exchange, autologous physiological renal replacement.	In vivo preclinical	[75,76,77,78]
		Ceramic-based iBAK Bioreactor	Qiu, J. et al.	Porous bioceramic membrane; perfused renal cell monolayer.	Selective filtration, selective solute/water reabsorption, reduction in systemic inflammatory cytokines.	In vivo preclinical	[79,80]

Abbreviations: WAK, wearable artificial kidney; AWAK, automated wearable artificial kidney; IHFD, implantable hemofiltration device; iBAK, implantable bioartificial kidney; RAD, renal assist device; BRECS, bioartificial renal epithelial cell system; SNMs, silicon nanopore membranes; TFI, thin film interposition.

**Table 2:** Major challenges and future strategies for BAK development.

Category	Critical Issue	Current Limitations	Possible Future Solutions or Research Priorities
Biological	Cell source availability and phenotype stability	Limited donor-derived renal cells; disease-associated cellular dysfunction; phenotype instability during expansion; insufficient maturation of stem cell-derived renal cells.	PSC-derived renal epithelial cells; optimized differentiation protocols; GMP-compliant cell manufacturing; standardized cell sources.
	Immunoisolation and immune compatibility	Limited long-term protection against immune mediators; incomplete characterization of chronic immune responses; insufficient validation of nanoporous immunoisolation systems.	Advanced nanoporous membranes; biomaterial surface modification; immune-compatible cell sources; long-term large-animal validation.
	Long-term cellular viability and functional maintenance	Loss of epithelial polarity; impaired cell adhesion; phenotype alteration under continuous perfusion; limited long-term metabolic activity.	Biomimetic extracellular microenvironment; optimized cell–material interaction; dynamic perfusion systems; tissue-engineered renal modules.
Engineering	Hemocompatibility and thrombosis prevention	Protein adsorption; platelet activation; coagulation cascade activation; limited long-term blood compatibility.	Antifouling biomaterials; anticoagulant surface modification; hemocompatible membrane design; long-term thrombogenicity assessment.
	Membrane fouling and filtration durability	Protein fouling; nanopore obstruction; progressive decline in filtration efficiency.	Antifouling membranes; optimized nanoporous architecture; surface engineering; self-regulating filtration systems.
	Device integration, implantation, and long-term stability	Complex implantation procedures; limited structural durability; insufficient long-term animal validation.	Miniaturized device architecture; durable biomaterials; improved implantation strategies; extended large-animal studies.
	Real-time monitoring and intelligent control	Lack of integrated biosensors; limited monitoring of cellular function and device performance.	Miniaturized biosensors; microelectronics integration; artificial intelligence-based monitoring; closed-loop control systems.
Translational	Manufacturing scalability and standardization	Limited large-scale cell expansion; complex fabrication processes; insufficient quality-control systems.	GMP-scale manufacturing platforms; standardized production protocols; scalable fabrication technologies.
	Regulatory approval and clinical translation	Unclear regulatory classification; complex safety evaluation; lack of predictive preclinical models; non-standardized clinical endpoints	Regulatory frameworks for biohybrid devices; validated large-animal models; standardized safety and efficacy criteria.
	Clinical outcomes, cost-effectiveness, and healthcare integration	Limited clinical evidence; insufficient patient-reported outcomes; unknown healthcare system impact.	Prospective clinical trials; quality-of-life assessment; economic evaluation; reimbursement models; healthcare integration strategies.

## Data Availability

No new data were created or analyzed in this study. Data sharing is not applicable.

## Abbreviations

The following abbreviations are used in this manuscript:

CKD	chronic kidney disease
ESRD	end-stage renal disease
RRT	renal replacement therapy
BAKs	bioartificial kidneys
iBAKs	implantable bioartificial kidneys
AI	artificial intelligence
WAKs	wearable artificial kidneys
PERVs	porcine endogenous retroviruses
RAD	renal assist device
AKI	acute kidney injury
CVVH	continuous veno-venous hemofiltration
CRRT	continuous renal replacement therapy
BRECS	bioartificial renal epithelial cell system
HRECs	human renal epithelial cells
EP	enhanced propagation
SS	septic shock
TMP	transmembrane pressure
MEMS	microelectromechanical systems
SNMs	silicon nanopore membranes
pSBMA	polysulfobetaine methacrylate
TFI	thin film interposition
HPTCs	human primary proximal tubular cells
hTERT	human telomerase reverse transcriptase
iPSCs	induced pluripotent stem cells
PTL	proximal tubular-like

## References

[1] Glassock R.J., Warnock D.G., Delanaye P. (2017). The Global Burden of Chronic Kidney Disease: Estimates, Variability and Pitfalls. Nat. Rev. Nephrol..

[2] Mark P.B., Stafford L.K., Grams M.E., Aalruz H., Abd ElHafeez S., Abdelgalil A.A., Abdulkader R.S., Abeywickrama H.M., Abiodun O.O., Abramov D. (2025). Global, Regional, and National Burden of Chronic Kidney Disease in Adults, 1990–2023, and Its Attributable Risk Factors: A Systematic Analysis for the Global Burden of Disease Study 2023. Lancet.

[3] Ortiz A., Lees J.S., Torra R., Stel V.S., Kramer A., Mark P.B. (2026). The Updated Global Burden of Chronic Kidney Disease: One Death Every 20 Seconds. Nephrol. Dial. Transplant..

[4] Wang L., Xu X., Zhang M., Hu C., Zhang X., Li C., Nie S., Huang Z., Zhao Z., Hou F.F. (2023). Prevalence of Chronic Kidney Disease in China: Results From the Sixth China Chronic Disease and Risk Factor Surveillance. JAMA Intern. Med..

[5] Wilson S., Mone P., Jankauskas S.S., Gambardella J., Santulli G. (2021). Chronic Kidney Disease: Definition, Updated Epidemiology, Staging, and Mechanisms of Increased Cardiovascular Risk. J. Clin. Hypertens..

[6] Rafferty Q., Stafford L.K., Vos T., Thomé F.S., Aalruz H., Abate Y.H., Abbafati C., ElHafeez S.A., Abedi A., Abiodun O.O. (2025). Global, Regional, and National Prevalence of Kidney Failure with Replacement Therapy and Associated Aetiologies, 1990–2023: A Systematic Analysis for the Global Burden of Disease Study 2023. Lancet Glob. Health.

[7] Thurlow J., Joshi M., Yan G., Norris K.C., Agodoa L.Y., Yuan C.M., Nee R. (2021). Global Epidemiology of End-Stage Kidney Disease and Disparities in Kidney Replacement Therapy. Am. J. Nephrol..

[8] Flythe J.E., Watnick S. (2024). Dialysis for Chronic Kidney Failure: A Review. JAMA.

[9] Bello A.K., Okpechi I.G., Osman M.A., Cho Y., Cullis B., Htay H., Jha V., Makusidi M.A., McCulloch M., Shah N. (2022). Epidemiology of Peritoneal Dialysis Outcomes. Nat. Rev. Nephrol..

[10] Jagdale A., Cooper D.K.C., Iwase H., Gaston R.S. (2019). Chronic Dialysis in Patients with End-stage Renal Disease: Relevance to Kidney Xenotransplantation. Xenotransplantation.

[11] United States Renal Data System (2023). 2023 USRDS Annual Data Report: Epidemiology of Kidney Disease in the United States.

[12] United States Renal Data System (2025). 2025 USRDS Annual Data Report: Epidemiology of Kidney Disease in the United States.

[13] Sallam L., Alsharif F., Abaalalaa S., Alakeely R., Abdullah Z., Alkhamis Z., Sindi N., Sharif L.S. (2022). Prevalence of Depression in Patients with End-Stage Renal Disease Undergoing Hemodialysis in Saudi Arabia: A Cross-Sectional Study. Belitung Nurs. J..

[14] Stel V.S., Boenink R., Astley M.E., Boerstra B.A., Radunovic D., Skrunes R., Ruiz San Millán J.C., Slon Roblero M.F., Bell S., Ucio Mingo P. (2024). A Comparison of the Epidemiology of Kidney Replacement Therapy between Europe and the United States: 2021 Data of the ERA Registry and the USRDS. Nephrol. Dial. Transplant..

[15] United States Renal Data System (2024). 2024 USRDS Annual Data Report: Epidemiology of Kidney Disease in the United States.

[16] Zoccali C., Mallamaci F., Lightstone L., Jha V., Pollock C., Tuttle K., Kotanko P., Wiecek A., Anders H.J., Remuzzi G. (2024). A New Era in the Science and Care of Kidney Diseases. Nat. Rev. Nephrol..

[17] Ibi Y., Nishinakamura R. (2023). Kidney Bioengineering for Transplantation. Transplantation.

[18] Yamaguchi T., Sato H., Kato-Itoh M., Goto T., Hara H., Sanbo M., Mizuno N., Kobayashi T., Yanagida A., Umino A. (2017). Interspecies Organogenesis Generates Autologous Functional Islets. Nature.

[19] Wu J., Platero-Luengo A., Sakurai M., Sugawara A., Gil M.A., Yamauchi T., Suzuki K., Bogliotti Y.S., Cuello C., Morales Valencia M. (2017). Interspecies Chimerism with Mammalian Pluripotent Stem Cells. Cell.

[20] Liu Q., Yue L., Deng J., Tan Y., Wu C. (2024). Progress and Breakthroughs in Human Kidney Organoid Research. Biochem. Biophys. Rep..

[21] Song J.J., Guyette J.P., Gilpin S.E., Gonzalez G., Vacanti J.P., Ott H.C. (2013). Regeneration and Experimental Orthotopic Transplantation of a Bioengineered Kidney. Nat. Med..

[22] Destefani A.C., Sirtoli G.M., Nogueira B.V. (2017). Advances in the Knowledge about Kidney Decellularization and Repopulation. Front. Bioeng. Biotechnol..

[23] Montgomery R.A., Stern J.M., Lonze B.E., Tatapudi V.S., Mangiola M., Wu M., Weldon E., Lawson N., Deterville C., Dieter R.A. (2022). Results of Two Cases of Pig-to-Human Kidney Xenotransplantation. N. Engl. J. Med..

[24] Porrett P.M., Orandi B.J., Kumar V., Houp J., Anderson D., Cozette Killian A., Hauptfeld-Dolejsek V., Martin D.E., Macedon S., Budd N. (2022). First Clinical-Grade Porcine Kidney Xenotransplant Using a Human Decedent Model. Am. J. Transplant..

[25] Stone L. (2023). Kidney Xenotransplantation. Nat. Rev. Urol..

[26] Nashan B. (2025). The First Clinical Renal Xenotransplantation Study (EXPAND): Evaluating Safety, Function, and Zoonotic Risks. Health Care Sci..

[27] Tao P., Zhou K. (2025). Recent Progress in Pig-to-Human Kidney Xenotransplantation. Front. Immunol..

[28] Tao K.-S., Yang Z.-X., Zhang X., Zhang H.-T., Yue S.-Q., Yang Y.-L., Song W.-J., Wang D.-S., Liu Z.-C., Li H.-M. (2025). Gene-Modified Pig-to-Human Liver Xenotransplantation. Nature.

[29] Denner J. (2024). Porcine Endogenous Retroviruses in Xenotransplantation. Nephrol. Dial. Transplant..

[30] Zidan A., El-Sherbini A.H., Noureldin A., Cooper D.K.C., Othman M. (2025). Characterizing Coagulation Responses in Humans and Nonhuman Primates Following Kidney Xenotransplantation—A Narrative Review. Am. J. Hematol..

[31] Garry M.G., Caplan A.L., Garry D.J. (2022). Emerging Technologies and Ethics—Exogenic Chimeric Humanized Organs. Am. J. Transplant..

[32] Humes H.D., Sobota J.T., Ding F., Song J.H. (2010). A Selective Cytopheretic Inhibitory Device to Treat the Immunological Dysregulation of Acute and Chronic Renal Failure. Blood Purif..

[33] Nikolovski J., Gulari E., Humes H.D. (1999). Design Engineering of a Bioartificial Renal Tubule Cell Therapy Device. Cell Transplant..

[34] Tumlin J., Wali R., Williams W., Murray P., Tolwani A.J., Vinnikova A.K., Szerlip H.M., Ye J., Paganini E.P., Dworkin L. (2008). Efficacy and Safety of Renal Tubule Cell Therapy for Acute Renal Failure. J. Am. Soc. Nephrol..

[35] Hodges S.J., Atala A. (2006). Initial Clinical Results of the Bioartificial Kidney Containing Human Cells in ICU Patients with Acute Renal Failure. Curr. Urol. Rep..

[36] Humes H.D., Fissell W.H., Weitzel W.F., Buffington D.A., Westover A.J., MacKay S.M., Gutierrez J.M. (2002). Metabolic Replacement of Kidney Function in Uremic Animals with a Bioartificial Kidney Containing Human Cells. Am. J. Kidney Dis..

[37] Humes H.D., Buffington D.A., MacKay S.M., Funke A.J., Weitzel W.F. (1999). Replacement of Renal Function in Uremic Animals with a Tissue-Engineered Kidney. Nat. Biotechnol..

[38] Humes H.D., Mackay S.M., Funke A.J., Buffington D.A. (1999). Tissue Engineering of a Bioartificial Renal Tubule Assist Device: In Vitro Transport and Metabolic Characteristics. Kidney Int..

[39] Fissell W.H., Roy S., Davenport A. (2013). Achieving More Frequent and Longer Dialysis for the Majority: Wearable Dialysis and Implantable Artificial Kidney Devices. Kidney Int..

[40] Johnston K.A., Westover A.J., Rojas-Pena A., Buffington D.A., Pino C.J., Smith P.L., Humes H.D. (2017). Development of a Wearable Bioartificial Kidney Using the Bioartificial Renal Epithelial Cell System (BRECS): A Wearable Kidney Using a BRECS. J. Tissue Eng. Regen. Med..

[41] Westover A.J., Buffington D.A., Johnston K.A., Smith P.L., Pino C.J., Humes H.D. (2017). A Bio-artificial Renal Epithelial Cell System Conveys Survival Advantage in a Porcine Model of Septic Shock. J. Tissue Eng. Regen. Med..

[42] Buffington D.A., Pino C.J., Chen L., Westover A.J., Hageman G., Humes H.D. (2012). Bioartificial Renal Epithelial Cell System (BRECS): A Compact, Cryopreservable Extracorporeal Renal Replacement Device. Cell Med..

[43] Gura V., Beizai M., Ezon C., Polaschegg H.-D., Ronco C. (2005). Continuous Renal Replacement Therapy for End-Stage Renal Disease. Contributions to Nephrology.

[44] Davenport A., Gura V., Ronco C., Beizai M., Ezon C., Rambod E. (2007). A Wearable Haemodialysis Device for Patients with End-Stage Renal Failure: A Pilot Study. Lancet.

[45] Gura V. (2021). Combination Wearable and Stationary Dialysis Systems. U.S. Patent.

[46] Gura V., Macy A.S., Beizai M., Ezon C., Golper T.A. (2009). Technical Breakthroughs in the Wearable Artificial Kidney (WAK). Clin. J. Am. Soc. Nephrol..

[47] Gura V., Davenport A., Beizai M., Ezon C., Ronco C. (2009). β2-Microglobulin and Phosphate Clearances Using a Wearable Artificial Kidney: A Pilot Study. Am. J. Kidney Dis..

[48] Gura V., Rivara M.B., Bieber S., Munshi R., Smith N.C., Linke L., Kundzins J., Beizai M., Ezon C., Kessler L. (2016). A Wearable Artificial Kidney for Patients with End-Stage Renal Disease. JCI Insight.

[49] Luo J., Yu H., Yang X., Wang D., Lu B., Liu J., Yang J., Zhang Y., Cheng S., Liu X. (2026). A Dialysate-Free Wearable Artificial Kidney Prototype Driven by a Liquid–Gas Phase Transition. Nat. Chem. Eng..

[50] Htay H., Gow S.K., Jayaballa M., Oei E.L., Chan C.-M., Wu S.-Y., Foo M.W. (2022). Preliminary Safety Study of the Automated Wearable Artificial Kidney (AWAK) in Peritoneal Dialysis Patients. Perit. Dial. Int..

[51] Bolhuis D., Bluechel C., Conan C., Ficheux M., Gerritsen K. (2025). First-in-Human Trial to Evaluate the Safety and Efficacy of Neokidney, a New Portable Hemodialysis Device. Nephrol. Dial. Transplant..

[52] Lau T.W., Bluechel C., Khaopaibul P., Tan H.J., Toh Y.E., Haroon S. (2024). First-in-Human Trial of the NeoKidney Portable Hemodialysis Device Sorbent System: FR-OR23. J. Am. Soc. Nephrol..

[53] Ficheux M., Bolhuis D.P., Conan C., Bluechel C., Gerritsen K.G. (2025). Clinical Assessment of Sodium and Acid-Base Balance with the Neokidney Sorbent-Based Portable Hemodialysis Device: FR-PO0532. J. Am. Soc. Nephrol..

[54] Zhou J. (2023). A Prospective, Cross-Over, Controlled Primary Clinical Evaluation of the Precision, Security, and Operability of Wearable Filtrating Artificial Kidney Device for On-Site Medical Rescue.

[55] To N., Sanada I., Ito H., Morita S., Kanno Y., Miki N. (2015). Development of Implantable Hemodialysis System Using PES Membranes with High Water-Permeability. Proceedings of the 2015 37th Annual International Conference of the IEEE Engineering in Medicine and Biology Society (EMBC), Milan, Italy, 25–29 August 2015.

[56] Ota T., To N., Kanno Y., Miki N. (2017). Evaluation of Biofouling in Stainless Microfluidic Channels for Implantable Multilayered Dialysis Device. Jpn. J. Appl. Phys..

[57] Ota T., Nakayama M., Kanno Y., Suzuki T., Miki N. (2018). In Vitro and in Vivo Tests of Nanoporous Membrane Coated with Biocompatible Fluorine-Doped Diamond-like Carbon for Hemofiltration Treatment. Proceedings of the 2018 IEEE Micro Electro Mechanical Systems (MEMS), Belfast, UK, 21–25 January 2018.

[58] Ito T., Ota T., Kono R., Miyaoka Y., Ishibashi H., Komori M., Yasukawa A., Kanno Y., Miki N. (2021). Pump-Free Microfluidic Hemofiltration Device. Micromachines.

[59] To N., Sanada I., Ito H., Prihandana G.S., Morita S., Kanno Y., Miki N. (2015). Water-Permeable Dialysis Membranes for Multi-Layered Microdialysis System. Front. Bioeng. Biotechnol..

[60] Kono R., Ota T., Kanno Y., Miki N. (2025). Design of Polyether Sulfone Flat-Sheet Membranes for Multi-Layered Hemodiafiltration Devices. PLoS ONE.

[61] Miki N., Ota T., Takasu M., Kanno Y. (2026). WCN26-7152 IMPLANTABLE ARTIFICIAL KIDNEY FOR HYBRID RENAL REPLACEMENT THERAPY. Kidney Int. Rep..

[62] Prihandana G.S., Sanada I., Ito H., Noborisaka M., Kanno Y., Suzuki T., Miki N. (2013). Antithrombogenicity of Fluorinated Diamond-Like Carbon Films Coated Nano Porous Polyethersulfone (PES) Membrane. Materials.

[63] ‘A Bridge to Transplant’: Implantable Dialysis Device Receives FDA Breakthrough Status. https://www.healio.com/news/nephrology/20260129/a-bridge-to-transplant-implantable-dialysis-device-receives-fda-breakthrough-status.

[64] Nephrodite Nephrodite Announces Industry’s First Successful Multi-Day Large Animal Study for a Fully Implantable Renal Replacement Device. https://nephrodite.com/newsroom/nephrodite-announces-industrys-first-successful-multi-day-large-animal/.

[65] Fissell W.H., Dubnisheva A., Eldridge A.N., Fleischman A.J., Zydney A.L., Roy S. (2009). High-Performance Silicon Nanopore Hemofiltration Membranes. J. Membr. Sci..

[66] Roy S., Dubnisheva A., Eldridge A., Fleischman A.J., Goldman K.G., Humes H.D., Zydney A.L., Fissell W.H. (2009). Silicon Nanopore Membrane Technology for an Implantable Artificial Kidney. Proceedings of the TRANSDUCERS 2009-2009 International Solid-State Sensors, Actuators and Microsystems Conference, Denver, CO, USA, 21–25 June 2009.

[67] Muthusubramaniam L., Lowe R., Fissell W.H., Li L., Marchant R.E., Desai T.A., Roy S. (2011). Hemocompatibility of Silicon-Based Substrates for Biomedical Implant Applications. Ann. Biomed. Eng..

[68] Kim S., Heller J., Iqbal Z., Kant R., Kim E.J., Durack J., Saeed M., Do L., Hetts S., Wilson M. (2016). Preliminary Diffusive Clearance of Silicon Nanopore Membranes in a Parallel Plate Configuration for Renal Replacement Therapy. ASAIO J..

[69] Kim S., Feinberg B., Kant R., Chui B., Goldman K., Park J., Moses W., Blaha C., Iqbal Z., Chow C. (2016). Diffusive Silicon Nanopore Membranes for Hemodialysis Applications. PLoS ONE.

[70] Kensinger C., Karp S., Kant R., Chui B.W., Goldman K., Yeager T., Gould E.R., Buck A., Laneve D.C., Groszek J.J. (2016). First Implantation of Silicon Nanopore Membrane Hemofilters. ASAIO J..

[71] Iqbal Z., Moses W., Kim S., Kim E.J., Fissell W.H., Roy S. (2018). Sterilization Effects on Ultrathin Film Polymer Coatings for Silicon-based Implantable Medical Devices. J. Biomed. Mater. Res. Part B Appl. Biomater..

[72] Iqbal Z., Kim S., Moyer J., Moses W., Abada E., Wright N., Kim E.J., Park J., Fissell W.H., Vartanian S. (2019). In Vitro and in Vivo Hemocompatibility Assessment of Ultrathin Sulfobetaine Polymer Coatings for Silicon-Based Implants. J. Biomater. Appl..

[73] Chen C., Santandreu A., Blaha C., Wright N., Moyer J., Kim E.J., Baltazar F.J., Chui B.W., Haniff T.M., Brakeman P.R. (2021). Demonstrating Preclinical Proof of Concept of an Implantable Bioartificial Kidney (iBAK): PO0513. J. Am. Soc. Nephrol..

[74] Kim E.J., Chen C., Gologorsky R., Santandreu A., Torres A., Wright N., Goodin M.S., Moyer J., Chui B.W., Blaha C. (2023). Feasibility of an Implantable Bioreactor for Renal Cell Therapy Using Silicon Nanopore Membranes. Nat. Commun..

[75] Klassen C.C. (2026). Thin Film Interposition of Basement Membrane Scaffolds.

[76] Klassen C.C., Ott H.C., Jaramillo M., Hesse R.G., Cheng D. (2022). Biological Fluid Purification with Biocompatible Membranes.

[77] Klassen C.C., Ford A., Singh M. (2025). Continuous Fiber Support Barrier for Engineered Vascular Networks.

[78] IVIVA Medical. https://www.ivivamedical.com/kidney-tissue-engineering.

[79] Chen Z., Zhao X., Zhuang G., Luo R., Zheng L., Wang H., Qiu J. (2024). Alumina Membrane, Preparation Method and Use Thereof.

[80] Qiu J., Xu C., Chen Z., Zheng L., Wang H., Li J. (2024). Implantable Artificial Kidney.

[81] Humes H.D., Buffington D., Westover A.J., Roy S., Fissell W.H. (2014). The Bioartificial Kidney: Current Status and Future Promise. Pediatr. Nephrol..

[82] Buffington D.A., Westover A.J., Johnston K.A., Humes H.D. (2014). The Bioartificial Kidney. Transl. Res..

[83] Ramada D.L., De Vries J., Vollenbroek J., Noor N., Ter Beek O., Mihăilă S.M., Wieringa F., Masereeuw R., Gerritsen K., Stamatialis D. (2023). Portable, Wearable and Implantable Artificial Kidney Systems: Needs, Opportunities and Challenges. Nat. Rev. Nephrol..

[84] Dominy C.L., Shamsian E.B., Okhawere K.E., Korn T.G., Meilika K., Badani K. (2023). Recent Innovations in Renal Replacement Technology and Potential Applications to Transplantation and Dialysis Patients: A Review of Current Methods. Kidney Res. Clin. Pract..

[85] Tang Y.-S., Tsai Y.-C., Chen T.-W., Li S.-Y. (2022). Artificial Kidney Engineering: The Development of Dialysis Membranes for Blood Purification. Membranes.

[86] Ip T.K., Aebischer P. (1989). Renal Epithelial-Cell-Controlled Solute Transport Across Permeable Membranes as the Foundation for a Bioartificial Kidney. Artif. Organs.

[87] Humes H.D., Weitzel W.F., Bartlett R.H., Swaniker F.C., Paganini E.P., Luderer J.R., Sobota J. (2004). Initial Clinical Results of the Bioartificial Kidney Containing Human Cells in ICU Patients with Acute Renal Failure. Kidney Int..

[88] Tasnim F., Deng R., Hu M., Liour S., Li Y., Ni M., Ying J.Y., Zink D. (2010). Achievements and Challenges in Bioartificial Kidney Development. Fibrogenes. Tissue Repair.

[89] Ronco C., Fecondini L. (2007). The Vicenza Wearable Artificial Kidney for Peritoneal Dialysis (ViWAK PD). Blood Purif..

[90] Himmelfarb J., Ratner B. (2020). Wearable Artificial Kidney: Problems, Progress and Prospects. Nat. Rev. Nephrol..

[91] Chircov C., Grumezescu A.M. (2022). Microelectromechanical Systems (MEMS) for Biomedical Applications. Micromachines.

[92] Moyer J., Wilson M.W., Sorrentino T.A., Santandreu A., Chen C., Hu D., Kerdok A., Porock E., Wright N., Ly J. (2023). Renal Embolization-Induced Uremic Swine Model for Assessment of Next-Generation Implantable Hemodialyzers. Toxins.

[93] Shi H.Y., Pang R., Yang J., Fan D., Cai H., Jiang H.B., Han J., Lee E.-S., Sun Y. (2022). Overview of Several Typical Ceramic Materials for Restorative Dentistry. Biomed. Res. Int..

[94] Vaiani L., Boccaccio A., Uva A.E., Palumbo G., Piccininni A., Guglielmi P., Cantore S., Santacroce L., Charitos I.A., Ballini A. (2023). Ceramic Materials for Biomedical Applications: An Overview on Properties and Fabrication Processes. J. Funct. Biomater..

[95] Bavya Devi K., Lalzawmliana V., Saidivya M., Kumar V., Roy M., Kumar Nandi S. (2022). Magnesium Phosphate Bioceramics for Bone Tissue Engineering. Chem. Rec..

[96] Li C., Sun W., Lu Z., Ao X., Li S. (2020). Ceramic Nanocomposite Membranes and Membrane Fouling: A Review. Water Res..

[97] Chen Z., Zhao X., Zhuang G., Luo R., Zheng L., Wang H., Qiu J. (2023). Preparation Method of Aluminum Oxide Film.

[98] Matai I., Kaur G., Seyedsalehi A., McClinton A., Laurencin C.T. (2020). Progress in 3D Bioprinting Technology for Tissue/Organ Regenerative Engineering. Biomaterials.

[99] Dai S., Wang Q., Jiang Z., Liu C., Teng X., Yan S., Xia D., Tuo Z., Bi L. (2022). Application of Three-Dimensional Printing Technology in Renal Diseases. Front. Med..

[100] Christou C.D., Vasileiadou S., Sotiroudis G., Tsoulfas G. (2023). Three-Dimensional Printing and Bioprinting in Renal Transplantation and Regenerative Medicine: Current Perspectives. J. Clin. Med..

[101] Mirkhalaf M., Men Y., Wang R., No Y., Zreiqat H. (2023). Personalized 3D Printed Bone Scaffolds: A Review. Acta Biomater..

[102] Syed Mohamed S.M.D., Welsh G.I., Roy I. (2023). Renal Tissue Engineering for Regenerative Medicine Using Polymers and Hydrogels. Biomater. Sci..

[103] Wieser M., Stadler G., Jennings P., Streubel B., Pfaller W., Ambros P., Riedl C., Katinger H., Grillari J., Grillari-Voglauer R. (2008). hTERT Alone Immortalizes Epithelial Cells of Renal Proximal Tubules without Changing Their Functional Characteristics. Am. J. Physiol. Ren. Physiol..

[104] Chandrasekaran V., Carta G., da Costa Pereira D., Gupta R., Murphy C., Feifel E., Kern G., Lechner J., Cavallo A.L., Gupta S. (2021). Generation and Characterization of iPSC-Derived Renal Proximal Tubule-like Cells with Extended Stability. Sci. Rep..

[105] Meijer T., Naderlinger E., Jennings P., Wilmes A. (2023). Differentiation and Subculturing of Renal Proximal Tubular-like Cells Derived from Human iPSC. Curr. Protoc..

[106] Joung Y.-H. (2013). Development of Implantable Medical Devices: From an Engineering Perspective. Int. Neurourol. J..

[107] Office of Combination Products About Combination Products. https://www.fda.gov/combination-products/about-combination-products.

[108] Salani M., Roy S., Fissell W.H. (2018). Innovations in Wearable and Implantable Artificial Kidneys. Am. J. Kidney Dis..

[109] Gray M., Meehan J., Ward C., Langdon S.P., Kunkler I.H., Murray A., Argyle D. (2018). Implantable Biosensors and Their Contribution to the Future of Precision Medicine. Vet. J..

[110] Alam F., Ashfaq Ahmed M., Jalal A.H., Siddiquee I., Adury R.Z., Hossain G.M.M., Pala N. (2024). Recent Progress and Challenges of Implantable Biodegradable Biosensors. Micromachines.

[111] Kim E.-S., Eun S.-J., Kim K.-H. (2023). Artificial Intelligence-Based Patient Monitoring System for Medical Support. Int. Neurourol. J..

[112] Leocadio P. (2024). Advancements in Implantable Medical Devices: A Comprehensive Analysis of Artificial Intelligence Integration, Adoption, and Applicability in Bio-Tech Innovations. Int. J. Comput. Sci. Inf. Technol..

